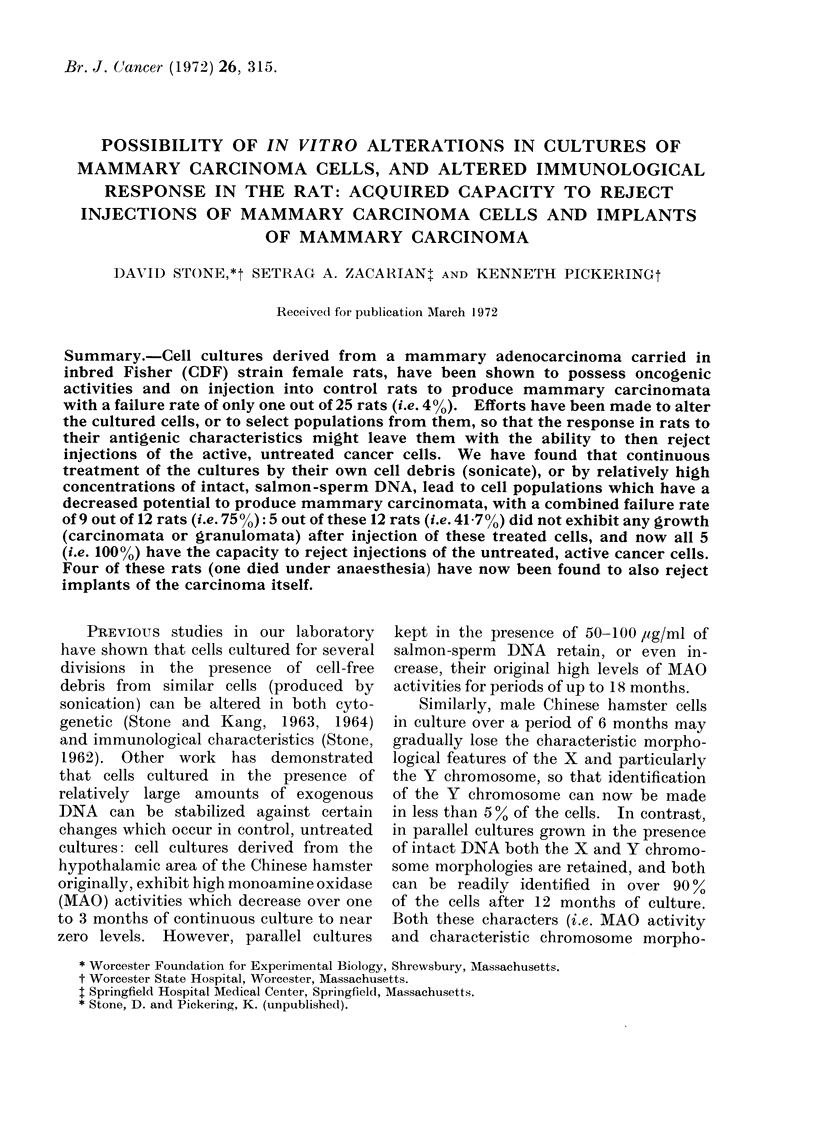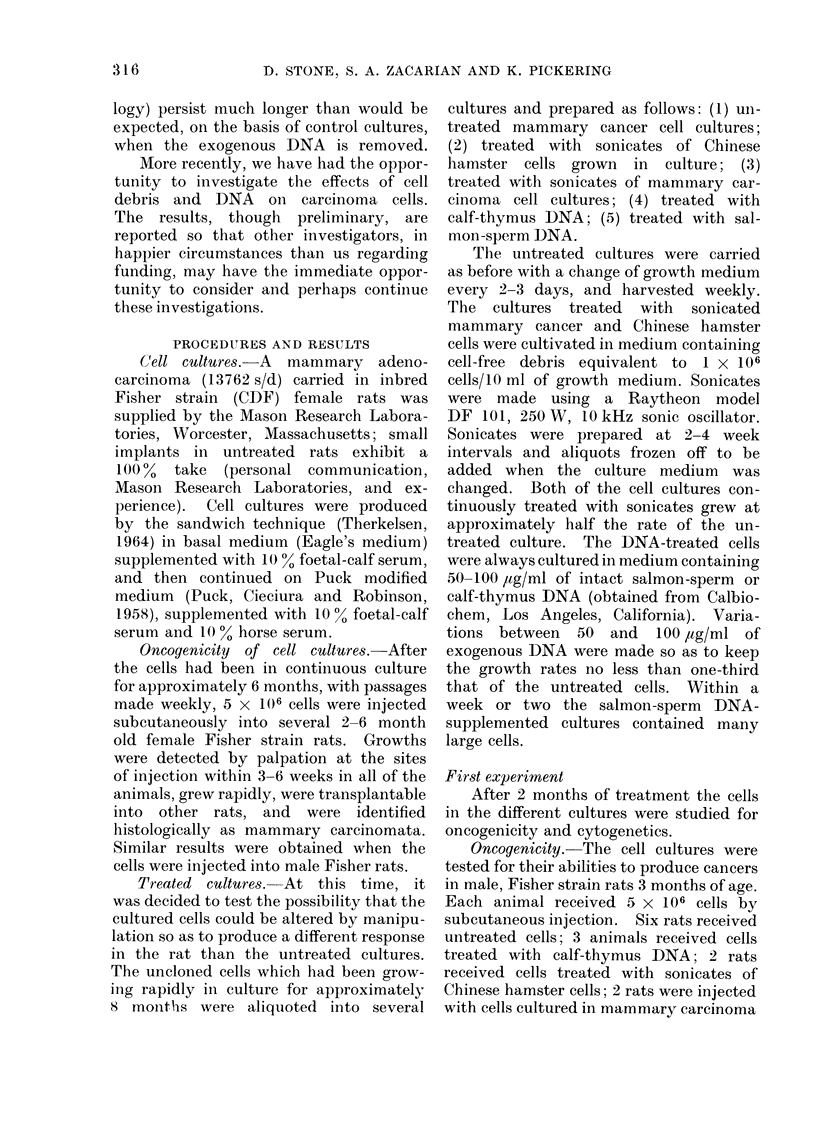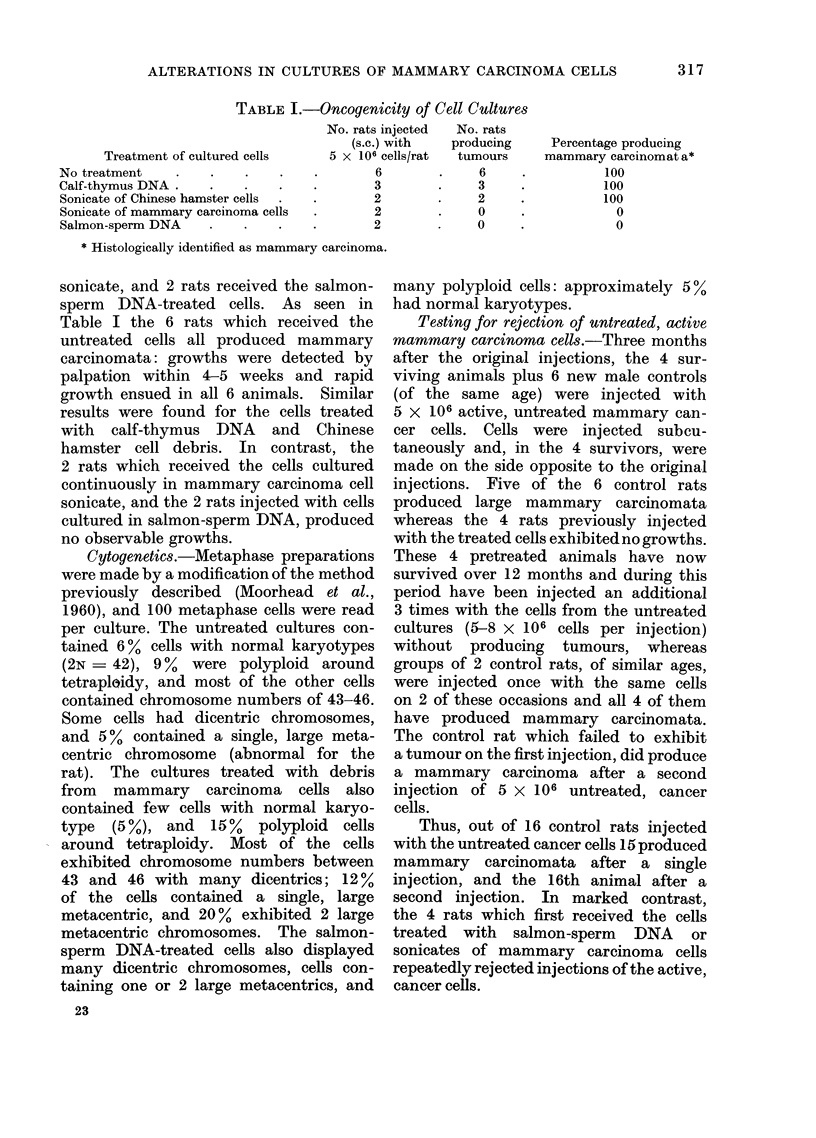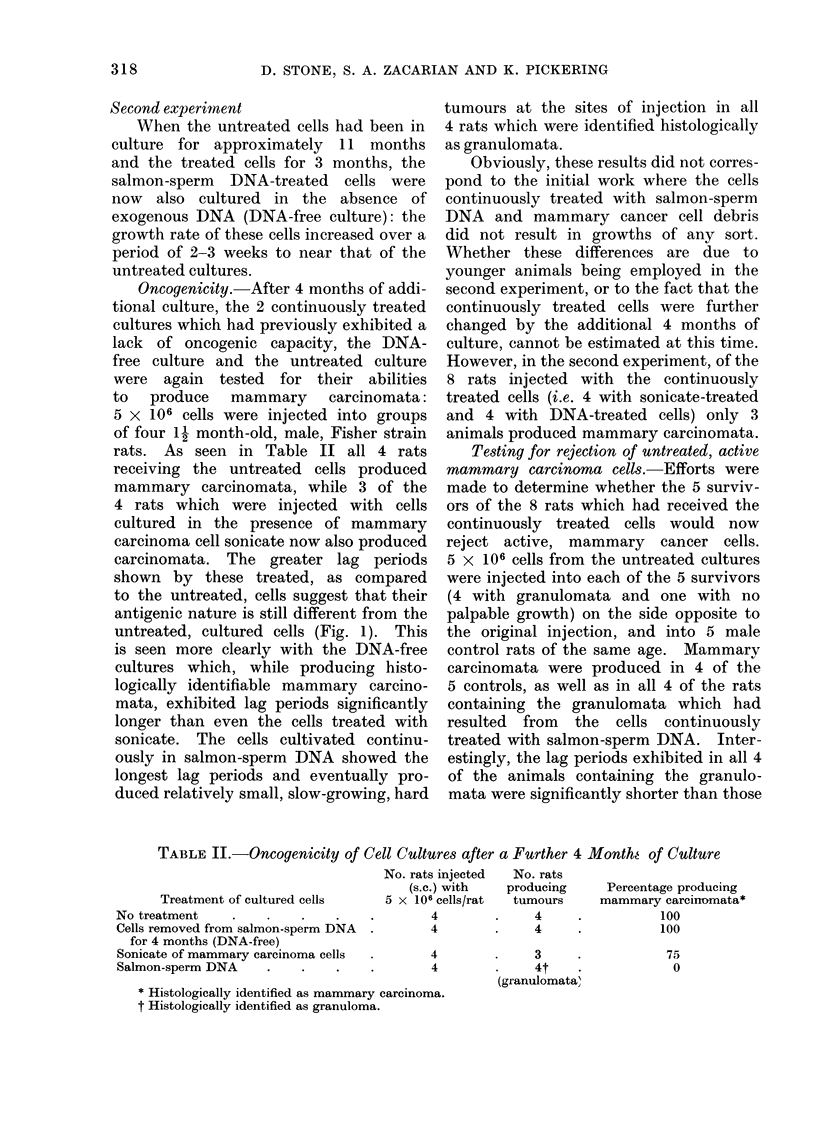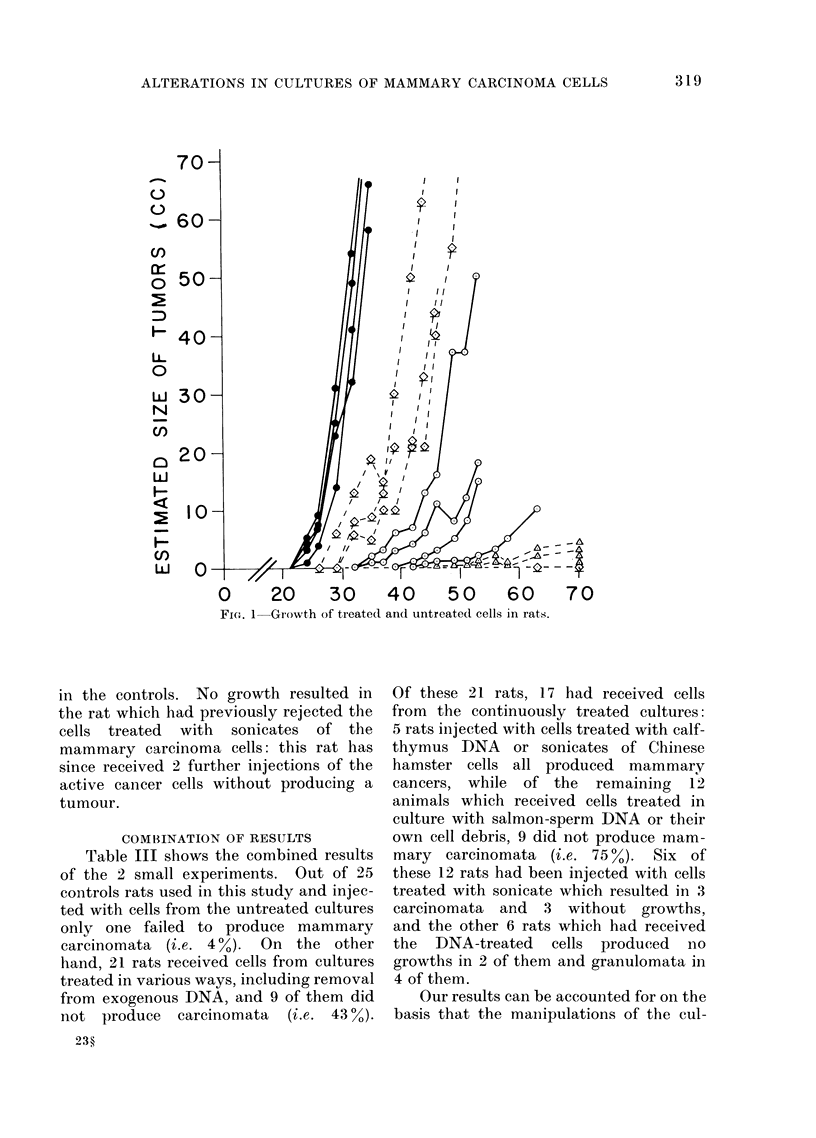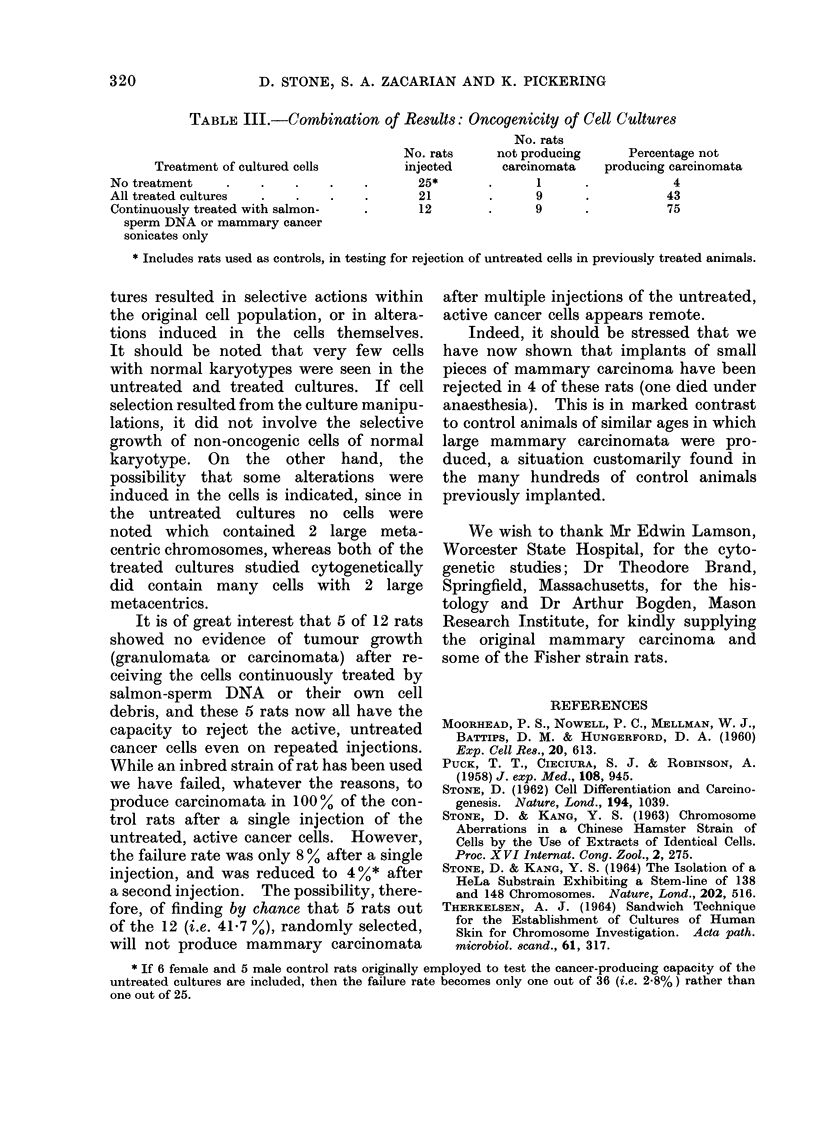# Possibility of in vitro Alterations in Cultures of Mammary Carcinoma Cells, and Altered Immunological Response in the Rat: Acquired Capacity to Reject Injections of Mammary Carcinoma Cells and Implants of Mammary Carcinoma

**DOI:** 10.1038/bjc.1972.42

**Published:** 1972-08

**Authors:** David Stone, Setrag A. Zacarian, Kenneth Pickering

## Abstract

Cell cultures derived from a mammary adenocarcinoma carried in inbred Fisher (CDF) strain female rats, have been shown to possess oncogenic activities and on injection into control rats to produce mammary carcinomata with a failure rate of only one out of 25 rats (*i.e.* 4%). Efforts have been made to alter the cultured cells, or to select populations from them, so that the response in rats to their antigenic characteristics might leave them with the ability to then reject injections of the active, untreated cancer cells. We have found that continuous treatment of the cultures by their own cell debris (sonicate), or by relatively high concentrations of intact, salmon-sperm DNA, lead to cell populations which have a decreased potential to produce mammary carcinomata, with a combined failure rate of 9 out of 12 rats (*i.e.* 75%): 5 out of these 12 rats (*i.e.* 41·7%) did not exhibit any growth (carcinomata or granulomata) after injection of these treated cells, and now all 5 (*i.e.* 100%) have the capacity to reject injections of the untreated, active cancer cells. Four of these rats (one died under anaesthesia) have now been found to also reject implants of the carcinoma itself.


Br. J. Cancer (1972) 26, 315.

### POSSIBILITY OF *IN VITRO* ALTERATIONS IN CULTURES OF MAMMARY CARCINOMA CELLS, AND ALTERED IMMUNOLOGICAL RESPONSE IN THE RAT: ACQUIRED CAPACITY TO REJECT INJECTIONS OF MAMMARY CARCINOMA CELLS AND IMPLANTS OF MAMMARY CARCINOMA

DAVID STONE,*† SETRAG A. ZACARIAN‡ AND KENNETH PICKERING†

Received for publication March 1972

**Summary.—Cell cultures derived from a mammary adenocarcinoma carried in inbred Fisher (CDF) strain female rats, have been shown to possess oncogenic activities and on injection into control rats to produce mammary carcinomata with a failure rate of only one out of 25 rats (*i.e.* 4%). Efforts have been made to alter the cultured cells, or to select populations from them, so that the response in rats to their antigenic characteristics might leave them with the ability to then reject injections of the active, untreated cancer cells. We have found that continuous treatment of the cultures by their own cell debris (sonicate), or by relatively high concentrations of intact, salmon-sperm DNA, lead to cell populations which have a decreased potential to produce mammary carcinomata, with a combined failure rate of 9 out of 12 rats (*i.e.* 75%): 5 out of these 12 rats (*i.e.* 41·7%) did not exhibit any growth (carcinomata or granulomata) after injection of these treated cells, and now all 5 (*i.e.* 100%) have the capacity to reject injections of the untreated, active cancer cells. Four of these rats (one died under anaesthesia) have now been found to also reject implants of the carcinoma itself.**

PREVIOUS studies in our laboratory have shown that cells cultured for several divisions in the presence of cell-free debris from similar cells (produced by sonication) can be altered in both cytogenetic (Stone and Kang, 1963, 1964) and immunological characteristics (Stone, 1962). Other work has demonstrated that cells cultured in the presence of relatively large amounts of exogenous DNA can be stabilized against certain changes which occur in control, untreated cultures: cell cultures derived from the hypothalamic area of the Chinese hamster originally, exhibit high monoamine oxidase (MAO) activities which decrease over one to 3 months of continuous culture to near zero levels. However, parallel cultures kept in the presence of 50–100 $\mu$g/ml of salmon-sperm DNA retain, or even increase, their original high levels of MAO activities for periods of up to 18 months.

Similarly, male Chinese hamster cells in culture over a period of 6 months may gradually lose the characteristic morphological features of the X and particularly the Y chromosome, so that identification of the Y chromosome can now be made in less than 5% of the cells. In contrast, in parallel cultures grown in the presence of intact DNA both the X and Y chromosome morphologies are retained, and both can be readily identified in over 90% of the cells after 12 months of culture. Both these characters (*i.e.* MAO activity and characteristic chromosome morpho-

* Worcester Foundation for Experimental Biology, Shrewsbury, Massachusetts.
† Worcester State Hospital, Worcester, Massachusetts.
‡ Springfield Hospital Medical Center, Springfield, Massachusetts.
* Stone, D. and Pickering, K. (unpublished).

logy) persist much longer than would be expected, on the basis of control cultures, when the exogenous DNA is removed.

More recently, we have had the opportunity to investigate the effects of cell debris and DNA on carcinoma cells. The results, though preliminary, are reported so that other investigators, in happier circumstances than us regarding funding, may have the immediate opportunity to consider and perhaps continue these investigations.

### PROCEDURES AND RESULTS

*Cell cultures.*—A mammary adenocarcinoma (13762 s/d) carried in inbred Fisher strain (CDF) female rats was supplied by the Mason Research Laboratories, Worcester, Massachusetts; small implants in untreated rats exhibit a 100% take (personal communication, Mason Research Laboratories, and experience). Cell cultures were produced by the sandwich technique (Therkelsen, 1964) in basal medium (Eagle's medium) supplemented with 10% foetal-calf serum, and then continued on Puck modified medium (Puck, Cieciura and Robinson, 1958), supplemented with 10% foetal-calf serum and 10% horse serum.

*Oncogenicity of cell cultures.*—After the cells had been in continuous culture for approximately 6 months, with passages made weekly, $5 \times 10^6$ cells were injected subcutaneously into several 2–6 month old female Fisher strain rats. Growths were detected by palpation at the sites of injection within 3–6 weeks in all of the animals, grew rapidly, were transplantable into other rats, and were identified histologically as mammary carcinomata. Similar results were obtained when the cells were injected into male Fisher rats.

*Treated cultures.*—At this time, it was decided to test the possibility that the cultured cells could be altered by manipulation so as to produce a different response in the rat than the untreated cultures. The uncloned cells which had been growing rapidly in culture for approximately 8 months were aliquoted into several cultures and prepared as follows: (1) untreated mammary cancer cell cultures; (2) treated with sonicates of Chinese hamster cells grown in culture; (3) treated with sonicates of mammary carcinoma cell cultures; (4) treated with calf-thymus DNA; (5) treated with salmon-sperm DNA.

The untreated cultures were carried as before with a change of growth medium every 2–3 days, and harvested weekly. The cultures treated with sonicated mammary cancer and Chinese hamster cells were cultivated in medium containing cell-free debris equivalent to $1 \times 10^6$ cells/10 ml of growth medium. Sonicates were made using a Raytheon model DF 101, 250 W, 10 kHz sonic oscillator. Sonicates were prepared at 2–4 week intervals and aliquots frozen off to be added when the culture medium was changed. Both of the cell cultures continuously treated with sonicates grew at approximately half the rate of the untreated culture. The DNA-treated cells were always cultured in medium containing 50–100 $\mu$g/ml of intact salmon-sperm or calf-thymus DNA (obtained from Calbiochem, Los Angeles, California). Variations between 50 and 100 $\mu$g/ml of exogenous DNA were made so as to keep the growth rates no less than one-third that of the untreated cells. Within a week or two the salmon-sperm DNA-supplemented cultures contained many large cells.

### *First experiment*

After 2 months of treatment the cells in the different cultures were studied for oncogenicity and cytogenetics.

*Oncogenicity.*—The cell cultures were tested for their abilities to produce cancers in male, Fisher strain rats 3 months of age. Each animal received $5 \times 10^6$ cells by subcutaneous injection. Six rats received untreated cells; 3 animals received cells treated with calf-thymus DNA; 2 rats received cells treated with sonicates of Chinese hamster cells; 2 rats were injected with cells cultured in mammary carcinoma

TABLE I.—*Oncogenicity of Cell Cultures*

| Treatment of cultured cells | No. rats injected (s.c.) with $5 \times 10^6$ cells/rat | No. rats producing tumours | Percentage producing mammary carcinomata* |
|---|---|---|---|
| No treatment | 6 | 6 | 100 |
| Calf-thymus DNA | 3 | 3 | 100 |
| Sonicate of Chinese hamster cells | 2 | 2 | 100 |
| Sonicate of mammary carcinoma cells | 2 | 0 | 0 |
| Salmon-sperm DNA | 2 | 0 | 0 |

* Histologically identified as mammary carcinoma.

sonicate, and 2 rats received the salmon-sperm DNA-treated cells. As seen in Table I the 6 rats which received the untreated cells all produced mammary carcinomata: growths were detected by palpation within 4–5 weeks and rapid growth ensued in all 6 animals. Similar results were found for the cells treated with calf-thymus DNA and Chinese hamster cell debris. In contrast, the 2 rats which received the cells cultured continuously in mammary carcinoma cell sonicate, and the 2 rats injected with cells cultured in salmon-sperm DNA, produced no observable growths.

*Cytogenetics.*—Metaphase preparations were made by a modification of the method previously described (Moorhead *et al.*, 1960), and 100 metaphase cells were read per culture. The untreated cultures contained 6% cells with normal karyotypes (2N = 42), 9% were polyploid around tetraploidy, and most of the other cells contained chromosome numbers of 43–46. Some cells had dicentric chromosomes, and 5% contained a single, large metacentric chromosome (abnormal for the rat). The cultures treated with debris from mammary carcinoma cells also contained few cells with normal karyotype (5%), and 15% polyploid cells around tetraploidy. Most of the cells exhibited chromosome numbers between 43 and 46 with many dicentrics; 12% of the cells contained a single, large metacentric, and 20% exhibited 2 large metacentric chromosomes. The salmon-sperm DNA-treated cells also displayed many dicentric chromosomes, cells containing one or 2 large metacentrics, and many polyploid cells: approximately 5% had normal karyotypes.

*Testing for rejection of untreated, active mammary carcinoma cells.*—Three months after the original injections, the 4 surviving animals plus 6 new male controls (of the same age) were injected with $5 \times 10^6$ active, untreated mammary cancer cells. Cells were injected subcutaneously and, in the 4 survivors, were made on the side opposite to the original injections. Five of the 6 control rats produced large mammary carcinomata whereas the 4 rats previously injected with the treated cells exhibited no growths. These 4 pretreated animals have now survived over 12 months and during this period have been injected an additional 3 times with the cells from the untreated cultures ($5–8 \times 10^6$ cells per injection) without producing tumours, whereas groups of 2 control rats, of similar ages, were injected once with the same cells on 2 of these occasions and all 4 of them have produced mammary carcinomata. The control rat which failed to exhibit a tumour on the first injection, did produce a mammary carcinoma after a second injection of $5 \times 10^6$ untreated, cancer cells.

Thus, out of 16 control rats injected with the untreated cancer cells 15 produced mammary carcinomata after a single injection, and the 16th animal after a second injection. In marked contrast, the 4 rats which first received the cells treated with salmon-sperm DNA or sonicates of mammary carcinoma cells repeatedly rejected injections of the active, cancer cells.

*Second experiment*

When the untreated cells had been in culture for approximately 11 months and the treated cells for 3 months, the salmon-sperm DNA-treated cells were now also cultured in the absence of exogenous DNA (DNA-free culture): the growth rate of these cells increased over a period of 2–3 weeks to near that of the untreated cultures.

*Oncogenicity.*—After 4 months of additional culture, the 2 continuously treated cultures which had previously exhibited a lack of oncogenic capacity, the DNA-free culture and the untreated culture were again tested for their abilities to produce mammary carcinomata: $5 \times 10^6$ cells were injected into groups of four $1\frac{1}{2}$ month-old, male, Fisher strain rats. As seen in Table II all 4 rats receiving the untreated cells produced mammary carcinomata, while 3 of the 4 rats which were injected with cells cultured in the presence of mammary carcinoma cell sonicate now also produced carcinomata. The greater lag periods shown by these treated, as compared to the untreated, cells suggest that their antigenic nature is still different from the untreated, cultured cells (Fig. 1). This is seen more clearly with the DNA-free cultures which, while producing histologically identifiable mammary carcinomata, exhibited lag periods significantly longer than even the cells treated with sonicate. The cells cultivated continuously in salmon-sperm DNA showed the longest lag periods and eventually produced relatively small, slow-growing, hard tumours at the sites of injection in all 4 rats which were identified histologically as granulomata.

Obviously, these results did not correspond to the initial work where the cells continuously treated with salmon-sperm DNA and mammary cancer cell debris did not result in growths of any sort. Whether these differences are due to younger animals being employed in the second experiment, or to the fact that the continuously treated cells were further changed by the additional 4 months of culture, cannot be estimated at this time. However, in the second experiment, of the 8 rats injected with the continuously treated cells (*i.e.* 4 with sonicate-treated and 4 with DNA-treated cells) only 3 animals produced mammary carcinomata.

*Testing for rejection of untreated, active mammary carcinoma cells.*—Efforts were made to determine whether the 5 survivors of the 8 rats which had received the continuously treated cells would now reject active, mammary cancer cells. $5 \times 10^6$ cells from the untreated cultures were injected into each of the 5 survivors (4 with granulomata and one with no palpable growth) on the side opposite to the original injection, and into 5 male control rats of the same age. Mammary carcinomata were produced in 4 of the 5 controls, as well as in all 4 of the rats containing the granulomata which had resulted from the cells continuously treated with salmon-sperm DNA. Interestingly, the lag periods exhibited in all 4 of the animals containing the granulomata were significantly shorter than those

TABLE II.—*Oncogenicity of Cell Cultures after a Further 4 Months of Culture*

| Treatment of cultured cells | No. rats injected (s.c.) with $5 \times 10^6$ cells/rat | No. rats producing tumours | Percentage producing mammary carcinomata* |
|---|---|---|---|
| No treatment | 4 | 4 | 100 |
| Cells removed from salmon-sperm DNA for 4 months (DNA-free) | 4 | 4 | 100 |
| Sonicate of mammary carcinoma cells | 4 | 3 | 75 |
| Salmon-sperm DNA | 4 | 4† (granulomata) | 0 |

* Histologically identified as mammary carcinoma.
† Histologically identified as granuloma.

FIG. 1—Growth of treated and untreated cells in rats.

in the controls. No growth resulted in the rat which had previously rejected the cells treated with sonicates of the mammary carcinoma cells: this rat has since received 2 further injections of the active cancer cells without producing a tumour.

COMBINATION OF RESULTS

Table III shows the combined results of the 2 small experiments. Out of 25 controls rats used in this study and injected with cells from the untreated cultures only one failed to produce mammary carcinomata (*i.e.* 4%). On the other hand, 21 rats received cells from cultures treated in various ways, including removal from exogenous DNA, and 9 of them did not produce carcinomata (*i.e.* 43%). Of these 21 rats, 17 had received cells from the continuously treated cultures: 5 rats injected with cells treated with calf-thymus DNA or sonicates of Chinese hamster cells all produced mammary cancers, while of the remaining 12 animals which received cells treated in culture with salmon-sperm DNA or their own cell debris, 9 did not produce mammary carcinomata (*i.e.* 75%). Six of these 12 rats had been injected with cells treated with sonicate which resulted in 3 carcinomata and 3 without growths, and the other 6 rats which had received the DNA-treated cells produced no growths in 2 of them and granulomata in 4 of them.

Our results can be accounted for on the basis that the manipulations of the cul-

TABLE III.—*Combination of Results: Oncogenicity of Cell Cultures*

| Treatment of cultured cells | No. rats injected | No. rats not producing carcinomata | Percentage not producing carcinomata |
|---|---|---|---|
| No treatment | 25* | 1 | 4 |
| All treated cultures | 21 | 9 | 43 |
| Continuously treated with salmon-sperm DNA or mammary cancer sonicates only | 12 | 9 | 75 |

* Includes rats used as controls, in testing for rejection of untreated cells in previously treated animals.

tures resulted in selective actions within the original cell population, or in alterations induced in the cells themselves. It should be noted that very few cells with normal karyotypes were seen in the untreated and treated cultures. If cell selection resulted from the culture manipulations, it did not involve the selective growth of non-oncogenic cells of normal karyotype. On the other hand, the possibility that some alterations were induced in the cells is indicated, since in the untreated cultures no cells were noted which contained 2 large metacentric chromosomes, whereas both of the treated cultures studied cytogenetically did contain many cells with 2 large metacentrics.

It is of great interest that 5 of 12 rats showed no evidence of tumour growth (granulomata or carcinomata) after receiving the cells continuously treated by salmon-sperm DNA or their own cell debris, and these 5 rats now all have the capacity to reject the active, untreated cancer cells even on repeated injections. While an inbred strain of rat has been used we have failed, whatever the reasons, to produce carcinomata in 100% of the control rats after a single injection of the untreated, active cancer cells. However, the failure rate was only 8% after a single injection, and was reduced to 4%* after a second injection. The possibility, therefore, of finding *by chance* that 5 rats out of the 12 (*i.e.* 41·7%), randomly selected, will not produce mammary carcinomata after multiple injections of the untreated, active cancer cells appears remote.

Indeed, it should be stressed that we have now shown that implants of small pieces of mammary carcinoma have been rejected in 4 of these rats (one died under anaesthesia). This is in marked contrast to control animals of similar ages in which large mammary carcinomata were produced, a situation customarily found in the many hundreds of control animals previously implanted.

We wish to thank Mr Edwin Lamson, Worcester State Hospital, for the cytogenetic studies; Dr Theodore Brand, Springfield, Massachusetts, for the histology and Dr Arthur Bogden, Mason Research Institute, for kindly supplying the original mammary carcinoma and some of the Fisher strain rats.

### REFERENCES

MOORHEAD, P. S., NOWELL, P. C., MELLMAN, W. J., BATTIPS, D. M. & HUNGERFORD, D. A. (1960) *Exp. Cell Res.*, **20**, 613.

PUCK, T. T., CIECIURA, S. J. & ROBINSON, A. (1958) *J. exp. Med.*, **108**, 945.

STONE, D. (1962) Cell Differentiation and Carcinogenesis. *Nature, Lond.*, **194**, 1039.

STONE, D. & KANG, Y. S. (1963) Chromosome Aberrations in a Chinese Hamster Strain of Cells by the Use of Extracts of Identical Cells. *Proc. XVI Internat. Cong. Zool.*, **2**, 275.

STONE, D. & KANG, Y. S. (1964) The Isolation of a HeLa Substrain Exhibiting a Stem-line of 138 and 148 Chromosomes. *Nature, Lond.*, **202**, 516.

THERKELSEN, A. J. (1964) Sandwich Technique for the Establishment of Cultures of Human Skin for Chromosome Investigation. *Acta path. microbiol. scand.*, **61**, 317.

* If 6 female and 5 male control rats originally employed to test the cancer-producing capacity of the untreated cultures are included, then the failure rate becomes only one out of 36 (*i.e.* 2·8%) rather than one out of 25.